# Molecular Detection and Genetic Characterization of Japanese Encephalitis Virus in Animals from 11 Provinces in China

**DOI:** 10.3390/v15030625

**Published:** 2023-02-24

**Authors:** Guanyu Zhao, Yan Gao, Ning Shi, Shiheng Zhang, Pengpeng Xiao, Jiaqi Zhang, Changzhan Xie, Zhuo Ha, Sheng Feng, Chenghui Li, Xuancheng Zhang, Yubiao Xie, Ning Yu, He Zhang, Junlong Bi, Ningyi Jin

**Affiliations:** 1 College of Veterinary Medicine, Jilin University, Changchun 130062, China; 2 Changchun Veterinary Research Institute, Chinese Academy of Agricultural Sciences, Changchun 130122, China; 3 Wenzhou Key Laboratory for Virology and Immunology, Institute of Virology, Wenzhou University, Wenzhou 325035, China; 4 College of Animal Veterinary Medicine, Yunnan Agricultural University, Kunming 650201, China

**Keywords:** molecular epidemiology, Japanese encephalitis virus, swine

## Abstract

Japanese encephalitis virus (JEV), which uses a mosquito primary vector and swine as a reservoir host, poses a significant risk to human and animal health. JEV can be detected in cattle, goats and dogs. A molecular epidemiological survey of JEV was conducted in 3105 mammals from five species, swine, fox, racoon dog, yak and goat, and 17,300 mosquitoes from 11 Chinese provinces. JEV was detected in pigs from Heilongjiang (12/328, 3.66%), Jilin (17/642, 2.65%), Shandong (14/832, 1.68%), Guangxi (8/278, 2.88%) and Inner Mongolia (9/952, 0.94%); in goats (1/51, 1.96%) from Tibet; and mosquitoes (6/131, 4.58%) from Yunnan. A total of 13 JEV envelope (E) gene sequences were amplified in pigs from Heilongjiang (5/13), Jilin (2/13) and Guangxi (6/13). Swine had the highest JEV infection rate of any animal species, and the highest infection rates were found in Heilongjiang. Phylogenetic analysis indicated that the predominant strain in Northern China was genotype I. Mutations were found at residues 76, 95, 123, 138, 244, 474 and 475 of E protein but all sequences had predicted glycosylation sites at ′N154. Three strains lacked the threonine 76 phosphorylation site from non-specific (unsp) and protein kinase G (PKG) site predictions; one lacked the threonine 186 phosphorylation site from protein kinase II (CKII) prediction; and one lacked the tyrosine 90 phosphorylation site from epidermal growth factor receptor (EGFR) prediction. The aim of the current study was to contribute to JEV prevention and control through the characterization of its molecular epidemiology and prediction of functional changes due to E-protein mutations.

## 1. Introduction

JEV causes epidemic encephalitis [1,2]. It has been reported in 24 countries, including Australia, Japan, India and most regions of the Western Pacific and Southeast Asia. Five genotypes, I–V, have been described based on the E-protein nucleotide sequence. Genotype I was discovered in Cambodia in 1967 [3] and coexists with genotype III, discovered in Japan and Vietnam during 1986–1990 [1,2], to become the most prevalent in Asia in the early to mid-1990s, when it was shown to be predominant in mosquitoes and swine [4]. Genotype I shows particularly efficient mosquito transmission with a wider and more rapid dispersal recorded in Japan [5].

The JEV natural transmission cycle includes a mosquito primary vector with swine as an amplifying host. Fourteen species of mosquito have been identified as JEV vectors [6], and the *Culex tritaeniorhynchus* subgroup within the *Culex vishnui* is the most significant [7]. Intensive swine farming in some parts of East and Southeast Asia facilitates the JEV transmission cycle [8], and industrialized farming increases transmission risk [9]. Pigs infected with JEV are usually asymptomatic or show only mild disease symptoms [10]. Pigs seldom show clinical signs. Other domesticated animals may also be infected by mosquito bites in endemic areas, although cattle, goats, dogs and foxes are thought to be dead-end hosts [11]. Pigs and dogs have been reported to give negative PCR tests but to have JEV-neutralizing antibodies [12]. Cattle and goats have been regarded as acceptable surveillance animals, due to the feeding preferences of vectors for these species, to enable risk assessment for human JEV infection [13].

Testing for JEV in China has focused on mosquitoes and swine with other domesticated animals being neglected, and this may present a potential risk. The current study performed a molecular epidemiological survey of JEV in foxes, minks, yaks and goats, in addition to swine and mosquitoes. The aim was to facilitate the prediction of JEV outbreaks in animals and human populations.

## 2. Materials and Methods

### 2.1. Animal Samples

A total of 3105 samples of pig serum were collected from farms in Heilongjiang (n = 328), Jilin (n = 642), Liaoning (n = 73), Shandong (n = 832) provinces, Guangxi (n = 278) and Inner Mongolia Autonomous Region (n = 952) between 2016 and 2020. Samples of fox serum (n = 719) were collected from fox farms in Jilin (n = 120), Liaoning (n = 267), Shandong (n = 234) and Hebei provinces (n = 98). A total of 13 yak and 51 goat samples were collected from Tibet. Racoon dog serum was obtained from Hebei (n = 132).

The *Culex tritaeniorhynchus* mosquito is the main JEV vector. Mosquito traps were placed outdoors 2 h after dusk and removed before dawn to collect and preserve all flying insects from sites near pig and cattle farms. Insects were examined under the microscope and mosquitoes were identified from compound eyes, antennae and proboscides (piercing sucking mouthparts). *Culex tritaeniorhynchus* mosquitoes were identified from their brown body, broad white ring around the middle part of the proboscis, white antennae tips, narrow white ring around the tarsus base and light-colored bands around the 2nd–7th abdominal segments and counted. Numbers of *Culex tritaeniorhynchus* collected were as follows: Jilin (n = 30), Liaoning (n = 4), Hubei (n = 1), Sichuan (n = 3), Tibet (n = 4) and Yunnan (n = 131), and 100 mosquitoes were considered to count as one sample. Samples were ground in RNase-free water at 4 °C, centrifuged and the supernatant filtered through a 0.22 µM filter to remove bacteria before storage at −80 °C.

### 2.2. Identification of Japanese Encephalitis Virus and Envelope Protein Sequence Amplification

Total RNA was extracted using a virus RNA extraction kit from Songon Biotech (SK1321, Shanghai, China) and reverse-transcribed into cDNA with a Reverse Transcription Kit from TaKaRa Biotechnology (RR036, Dalian, China). Primers (JEV ID) were designed to recognize the conserved region of JEV NS1 protein: forward 5′-TTTGGGGGGATGGTGTTGA-3′; reverse 5′-GCACCAGTCAGTGATCAACTTTCC-3′. PCR was performed with 1 µL of forward primer, 1 µL of reverse primer, 1 µL (2.5 mmol/L) of dNTPs, 4 µL of PCR buffer, 1 µL of sample cDNA, 1 µL of DNA polymerase (TransGen Biotech, Beijing, China) and 11 µL of nuclease-free water in a total volume of 20 μl. Samples were denatured at 94 °C for 5 min, followed by 35 cycles of denaturation at 95 °C for 30 s, annealing at 55 °C for 30 s and elongation at 72 °C for 30 s and a final 5 min elongation period with cooling to 4 °C. JEV envelope (E) protein sequence amplification primers (JEV E) were designed with reference to GenBank (www.ncbi.nlm.nih.gov/genbank/ (accessed on 2 June 2020)): forward 5′-GTTGACACCCATCCAAAGAAGTAAGGC-3′; reverse 5′-GTTGACACCCATCCAAAGAAGTAAGGC-3′. PCR was performed as JEV ID above. PCR products were visualized via gel electrophoresis with 1.5% agarose concentration, and target bands were cut out and purified with a Songon Biotech gel extraction kit. Sequencing was conducted by Comate Bioscience Co., Ltd. (Jilin, China).

### 2.3. Sequence Analysis

JEV nucleic acid sequences were retrieved from the National Center for Biotechnology Information (NCBI, https://www.ncbi.nlm.nih.gov/ (accessed on 15 December 2022)) [14] and 31 JEV genome sequences with five genotypes [15] from GenBank. Genome sequences included 10 genotype I samples: JN381830, JF706268, JN381831, JF706274, HM366552, JF706286, JF706281, JF706270, HQ652538 and DQ404128; 3 genotype II samples: U70406, U70421 and AF217620; 8 genotype III samples: KP164498, KF297915, JN381867, JN381856, JN381857, JF706276, JF706273 and JN381859; 6 genotype IV samples: U70407, U70409, LC461961, JQ429309, AY184212 and U70408 and 4 genotype V samples: JF915894, MF526900, HM596272 and KM677246. All sequences are given in Table 1. Nucleotide and predicted amino acid sequences were compiled and edited using the BioEdit (version 7.2) program and aligned using the MegAlign software (version 17.1) from ClustalW [16]. Phylogenetic analysis of nucleotide sequences (nt) and protein gene dataset was performed using MEGA version 6.0 maximum compound likelihood nucleotide substitution model. The robustness of the system diagram was evaluated via 1000 bootstrap copies.

### 2.4. Predicted N-Glycosylation and Phosphorylation Sites in JEV E Protein

N-glycosylation sites were predicted by NetNGlyc4.0Server (https://www.cbs.dtu.dk/services/NetNGlyc/ (accessed on 18 December 2022)) and phosphorylation sites by NetPhos 3.1Server (https://www.cbs.dtu.dk/services/NetPhos/ (accessed on 18 December 2022)).

### 2.5. Nucleotide Sequence Accession Numbers

Envelope protein sequences of 13 JEV strains have been deposited in the GenBank database (numbers: OP699289 to OP699301). All sequences were from swines: OP699289–OP699294 from Guangxi, China; OP699295–OP699299 from Heilongjiang, China; OP699300 and OP699301 from Jilin, China.

## 3. Results

### 3.1. Samples

**Figure 1:**
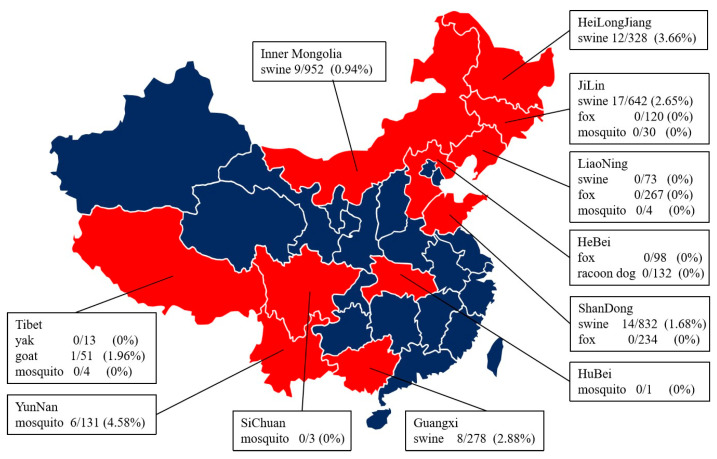
Sample collection information, including the location, type, number of samples, number of JEV positives and probability of JEV positivity in this study. Samples were collected from 11 provinces in China; 3105 swines, 17,300 mosquitoes, 51 goats, 13 yaks, 719 foxes and 179 racoon dogs were detected. Jilin Province had the largest number of JEV-positive cases, and Heilongjiang Province had the highest JEV-positive rate.

Samples from 17,300 mosquitos, 3105 pigs, 51 goats, 13 yaks, 719 foxes and 179 racoon dogs were tested for JEV between 2016 and 2020 (Figure 1). A total of 6/169 (3.55%) were positive for *Culex tritaeniorhynchus* in all provinces. Altogether, 60/3105 (1.93%) pigs gave positive results. Thirteen JEV envelope protein sequences were detected in swines; sequences OP699289 to OP699294 were detected in Guangxi, sequences OP699295 to OP699299 were detected in Heilongjiang and sequences OP699300 to OP699301 were detected in Jilin. A total of 1/51 (1.96%) goats, 0/132 (0.00%) racoon dogs and 0/13 (0.00%) yaks gave positive results. Mosquitoes gave the highest proportion of JEV-positive results (3.55%) of all samples, and pigs had the highest positive rate among mammals.

### 3.2. Phylogenetic Analysis of JEV

**Figure 2:**
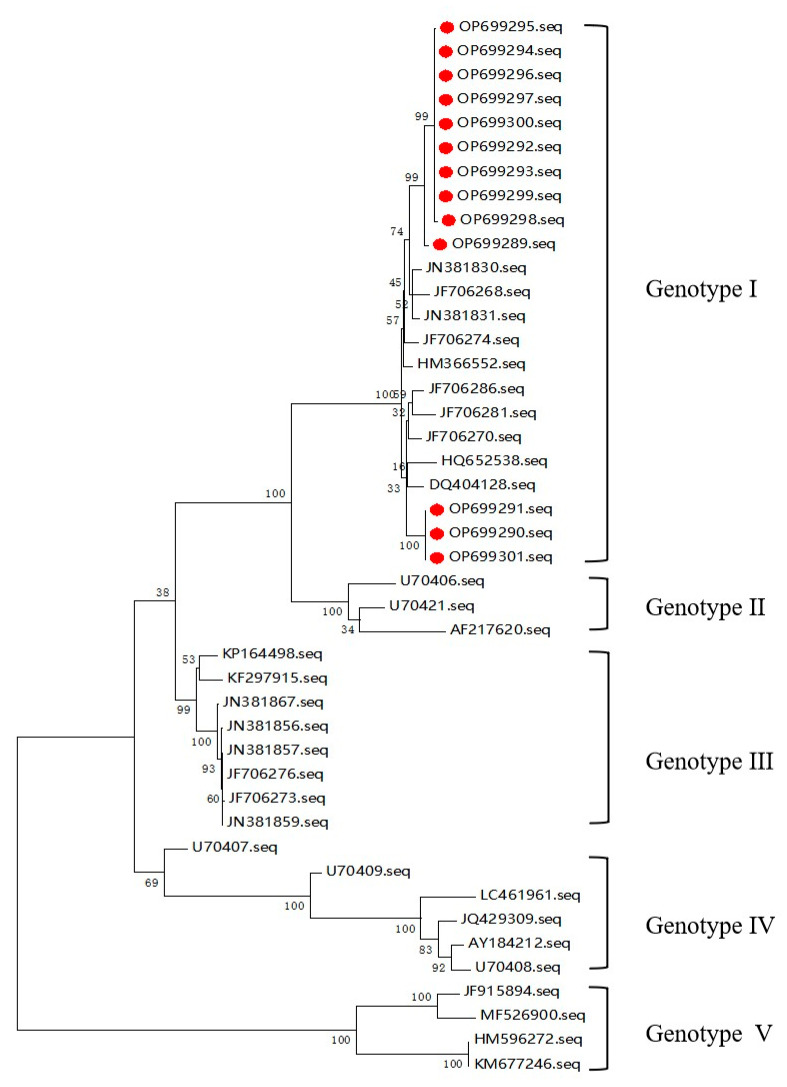
Maximum likelihood method produces a phylogenetic tree to determine the genotype of the detected JEV, red dot representative sequences detected in this study. All detected JEVs belong to genotype I. OP699289 and OP699292–OP699300 belong to the same branch, and they were more closely related to JN381830 and JF706268. OP699290, OP699291 and OP699301 belong to the same branch, and they were more closely related to DQ404128 and HQ652538. Sequences OP699289 to OP699294 were detected in Guangxi, sequences OP699295 to OP699299 were detected in Heilongjiang and sequences OP699300 to OP699301 were detected in Jilin.

Thirteen JEV E gene sequences were amplified from positive samples from pigs, and phylogenetic analysis revealed that all could be classified into genotype I clusters (Figure 2). OP699289 and OP699292–OP699300 were closely related to JN381830 and JF706268. OP699290, OP699291 and OP699300 were closely related to HQ652538 and DQ404128.

### 3.3. Alterations in Amino Acid Sequences

Predicted amino acid sequences were compared with the reference genome. OP699290 (Guangxi), OP699291 (Guangxi) and OP699301 (Jilin) all had mutations at residues 76, 244 and 475. Other strains had mutations at positions 95, 123, 138, 474 and 475 (Table 2).

### 3.4. Glycosylation and Phosphorylation Sites

**Figure 3:**
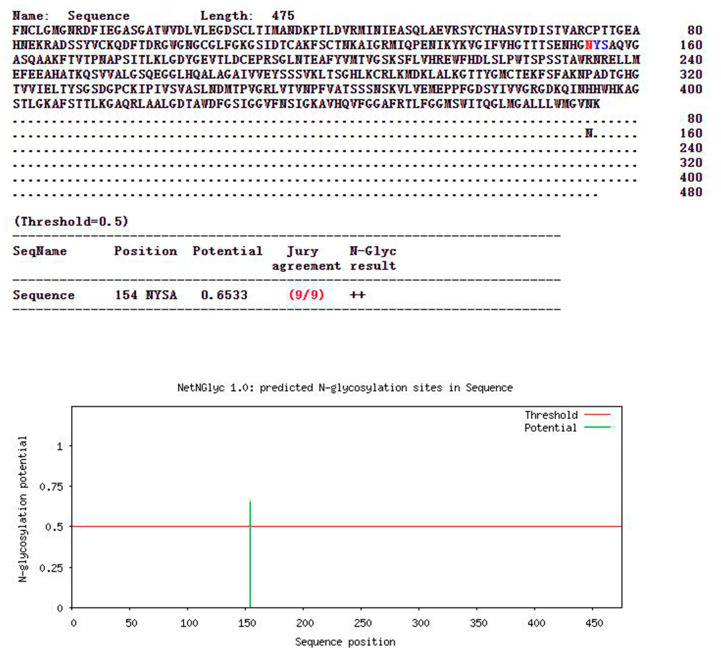
The ′N-glycosylation sites of 13 strains indicate the encoded JEV E proteins. All JEV strains had potential ′N-glycosylation sites at ′N154 with a confidence level of 9/9.

**Figure 4:**
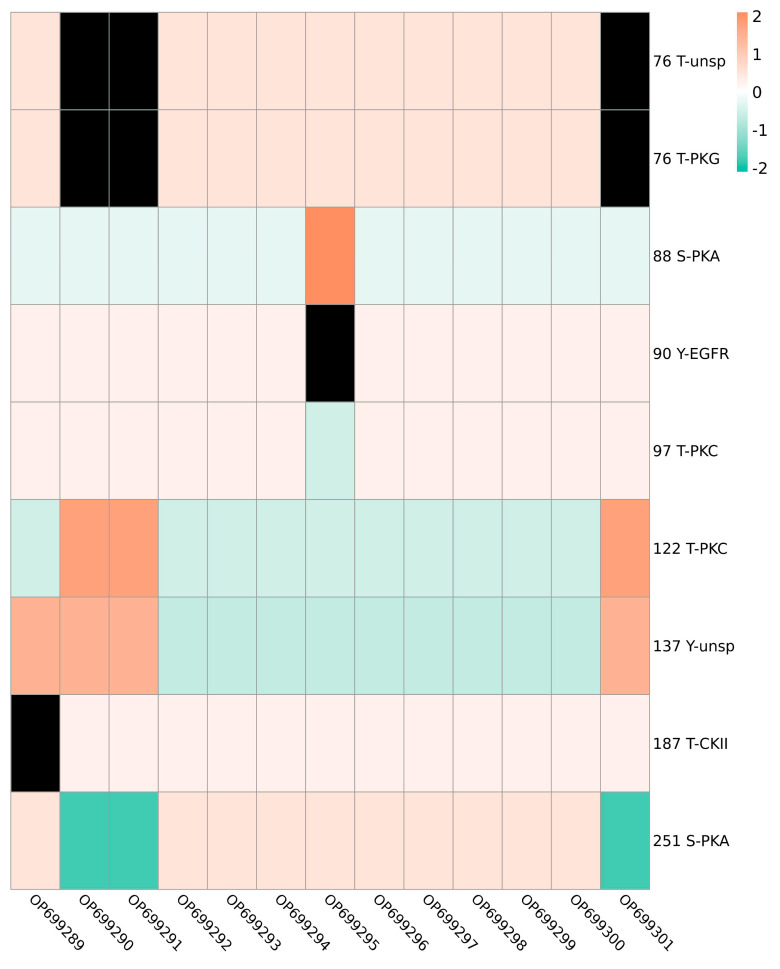
Unique phosphorylation sites in JEV strains. OP699290, OP699291 and OP699301 lacked phosphorylation sites at the 76th threonine with kinase predictions of unsp and PKG. At the 122nd threonine and 137th tyrosine phosphorylation sites, higher scores were attained than other strains, and kinase predictions of PKC and unsp, respectively, were improved. OP699289 lacked a phosphorylation site at the 187th threonine and CKII kinase was predicted. It was scored in line with OP699290, OP699291 and OP699301 at the 137th tyrosine. OP699295 had a higher score at the 88th serine phosphorylation site and lacked a phosphorylation site at the 90th tyrosine, and so the kinase was predicated to be an EGFR. The more orange represents a higher predictive value, the more green represents a low predictive value and black represents no phosphorylation site.

Gene sequences were converted into predicted amino acid sequences using Biosoft and the ′N-glycosylation sites of envelope proteins were predicted using NetNGlyc1.0Server. All 13 sequences isolated during the present work and 20 reference genotype I strains had a possible ′N-glycosylation site at position ′N154 (Figure 3). The same site was present in the JEV vaccine strains SA14-14-2 and BJ-1, indicating that this site remains constant across genotypes and strains. Phosphorylation sites were predicted using the NetPhos3.1Server, and 72–74 potential sites were identified in E proteins. All JEV strains had 66 identical phosphorylation sites (Table 3). OP699289 had 73 potential phosphorylation sites, including 35 on serine (S), 31 on threonine (T) and 9 on tyrosine (Y), and tyrosine 137 (Y) was a non-specific prediction (unsp). OP699290, OP699291 and OP699301 had more phosphorylation sites than other strains, but lacked phosphorylation at the threonine 187 site (Figure 4). OP699290, OP699291 and OP699301 had 72 potential phosphorylation sites, including 35 on serine, 30 on threonine and 9 on tyrosine and lacked phosphorylation on threonine 76 with non-specific prediction (unsp) and PKG. These three strains had higher positive prediction scores at threonine 122 with PKC and lower scores at serine 251 with PKA. OP699295 had 73 potential phosphorylation sites, including 35 on serine, 32 on threonine and 8 on tyrosine, with a higher positive prediction score at serine 88 with PKA. No phosphorylation site was seen at tyrosine 90 with EGFR kinase. OP699292, OP699293, OP699294, OP699296, OP699297, OP699298, OP699299 and OP699300 had 74 potential phosphorylation sites, including 35 on serine, 32 on threonine and 9 on tyrosine. The occurrence of such phosphorylation sites raises the possibility that JEV E protein is involved in immune control and viral replication.

## 4. Discussion

A total of 3105 mammals of five species and 17,300 mosquitoes were collected for epidemiological characterization. JEV was detected in pigs from the Heilongjiang, Jilin, Shandong and Guangxi provinces and in mosquitoes from Yunnan Province with JEV genotype I showing dominance. It has been suggested that genotype I JEV has become the dominant strain in China since its isolation in Yunnan in 1977 and that it coexists with genotype Ⅲ JEV. Genotype Ⅲ JEV was not detected during the current study, but a 2016 epidemiological survey in Zhejiang Province, China, showed it to be the second most prevalent strain after genotype I. Previous studies have suggested the division of genotype I into two branches, one of which persists in tropical Asia and the other of which has spread to China [17]. Genotype I is predominant in Thailand, Laos, Vietnam and Myanmar and was shown to persist in Japan and Thailand by a 2016–2018 survey, which may explain its detection in both the northern and southwestern provinces of China. Samples from the central East–West corridor were unavailable during the current study, but collection and analysis of JEV samples from this region were planned. Areas with a high JEV-positive rate were found to be ones where rice was cultivated, and so this may influence the spread of JEV. Ningxia had not experienced any reported JEV outbreaks in the 10 years prior to rice being planted, after which the 2018 JEV outbreak among humans was recorded [18]. JEV was detected in the northern and southwestern provinces of China at a lower-than-expected rate, probably due to the promotion of the anti-JEV swine vaccine and improvements in disease control in intensive feedlot swine farms. The sequences we detected in both phylogenetic clusters were almost identical sequences from two very disparate areas and in two different years. We speculate that it may have a slow mutation rate and connections between pigs from several provinces. We will be conducting more research in the future. International, trans-regional and multi-species JEV transmission remains a threat; JEV not only exists southeast of China but is also occasionally epidemic further north in maritime Siberia [19], and surveillance should not be neglected in China.

Swine, goats, yaks, foxes, racoon dogs and mosquitoes were all investigated, and swine, goats and mosquitos were found to be positive for JEV. The positive goat sample originated in Tibet, but more detailed information was unavailable. The diversity of JEV hosts reflects the adaptability of the virus and the feeding habits of mosquitoes [20]. *Culex tritaeniorhynchus* prefers cattle and swine [20], and since yaks and goats are often kept by the same village farmer or in free-range areas, interspecies transmission is favored. No JEV was isolated from mosquitoes in Tibet during the current study, which would have enabled a complete chain of multi-species transmission to be constructed. Previous studies have found JEV in pigs and mosquitoes in Tibet [21,22], suggesting multi-species transmission in areas of high altitude (over 3100 m above sea level). JEV was not detected in foxes, raccoon dogs or yaks until now. Humans, horses, dogs and cattle are considered dead-end hosts [23,24,25]. The ratio of dogs to humans may be 1:3 in some areas of the Philippines, increasing the risk of human infection [12], and captive racoon dogs and foxes exacerbate the situation. Surveys of sentinel animals help us to understand the spread of the virus, and future studies on other host species are planned. Wider studies on interspecies transmission are expected to further our understanding and ability to control the spread to humans. 

The JEV envelope glycoprotein is an antigenic protein that attaches to cell surface receptors to trigger antibody production [26,27]. Mutations in OP699290, OP699291 and OP699301 were found in the current study to be E244Q, T76M, E244Q and A475K. Mutations found in other strains were S123N, E138K, A475K and A475R. Residue 244 was normally the acidic amino acid, glutamic acid, and when mutated to the amphoteric amino acid, glycine [28], the infective capacity was weakened. Most JEV strains were found to have the E138K mutation, which is acknowledged to reduce the neurotoxicity of JEV [29]. We conclude that the 10 JEV strains of the present study exhibit low neurotoxicity and the other 3 the original neurotoxic capacity.

All JEV strains analyzed had a glycosylation site at position ′N154, as did the vaccine strains, SA14-14-2 and BJ-1. Glycosylated proteins promote the correct folding of proteins, enhance glycoprotein stability and are more resistant to proteases. Modifications to glycosylation patterns allow the identification of proteins for sorting and packaging in the Golgi apparatus for targeted transport. The glycosylation site of JEV E protein has been found to be essential for virus replication, infectivity, pathogenicity and neurotoxicity [30], and its cytotoxicity depends on its correct folding and assembly. Modification of the ′N154 site greatly improved the triggered humoral immune response to vaccine strains [31]. The JEV strains examined during the current study have retained this immunogenic capacity, which is good news for JEV control. Phosphorylation affects viral amplification [32], and E-protein phosphorylation sites were found to be very stable among strains. OP699290, OP699291 and OP699301 showed deletion of the 76T phosphorylation site and decreased phosphorylation at the 251S position, perhaps due to mutation of the amino acid sites. These changes may increase or decrease the neurotoxicity of the viral strains. More information is required to clarify the impact of the phosphorylation site deletion at position 187T of OP699289 and the 90Y deletion of OP699295.

We acknowledge some limitations to the present study. We demonstrate the expansion of the active range of JEV in both the northern and southwestern provinces of China. A greater range of geographical areas and JEV host ranges should be included in future epidemiological investigations. Migratory birds may be responsible for carrying the virus from tropical to temperate zones due to increased exposure to JEV vectors and may account for seasonal variations in infection waves. Human activities, such as international travel by land or sea, may also contribute to the expansion of the JEV infection range. In addition, the wide range of potential JEV hosts may contribute to increasing infection rates. Detailed information on multi-host infections during epidemiological studies is required. Additional systematic studies on mutations and their impact on JEV protein function are also merited.

In conclusion, the frequency of JEV in various Chinese provinces is reported, with Heilongjiang, Jilin and Inner Mongolia having the highest infection rates and pigs being the most likely animals to be JEV-positive. JEV genotype Ⅰ currently predominates in the north. Geographical and host range expansion of the virus makes JEV epidemics more likely, and more extensive molecular epidemiological studies are required to reduce biosecurity risks. Five JEV strains had mutations in phosphorylation sites. Detection of mutations and predictive analysis of glycosylation and phosphorylation sites provide insights for vaccine development.

## Figures and Tables

**Table 1:** Sequences used in this study.

Gene Number	Genotype	Origin of Species	Location	Sampling Time
JN381830	I	Mosquito	Henan, China	2006
JF706268	I	Mosquito	Yunnan, China	2009
JN381831	I	Mosquito	Henan, China	2004
JF706274	I	Mosquito	Gansu, China	2008
HM366552	I	Mosquito	Guizhou, China	2006
JF706286	I	Mosquito	Shandong, China	2008
JF706281	I	Mosquito	Yunnan, China	2005
JF706270	I	Mosquito	Guangxi, China	2006
HQ652538	I	Mosquito	Tibet, China	2009
DQ404128	I	Mosquito	Yunnan, China	1979
U70406	Ⅱ	Mosquito	Bali, Indonesia	1981
U70421	Ⅱ	Mosquito	Kuala Lumpur, Malaysia	1970
AF217620	Ⅱ	Human	Australia	1995
KP164498	Ⅲ	Swine	India	2014
KF297915	Ⅲ	Swine	Guangdong, China	2009
JN381867	Ⅲ	Human	Fujian, China	2002
JN381856	Ⅲ	Swine	Shanghai, China	2004
JN381857	Ⅲ	Mosquito	Guizhou, China	2004
JF706276	Ⅲ	Mosquito	Heilongjiang, China	2002
JF706273	Ⅲ	Swine	Fujian, China	2002
JN381859	Ⅲ	Human	Fujian, China	2003
U70407	Ⅳ	Mosquito	Indonesia	1981
U70409	Ⅳ	Mosquito	Indonesia	1981
LC461961	Ⅳ	Swine	Bali, Indonesia	2017
JQ429309	Ⅳ	Mosquito	Bantul, Indonesia	1981
AY184212	Ⅳ	Mosquito	Indonesia	1981
U70408	Ⅳ	Mosquito	Indonesia	1981
JF915894	V	Mosquito	Tibet, China	2009
MF526900	V	Mosquito	South Korea	2015
HM596272	V	Human	Malaysia	1952
KM677246	V	Human	Singapore	1952
OP699289	I	Swine	Guangxi, China	2016
OP699290	I	Swine	Guangxi, China	2016
OP699291	I	Swine	Guangxi, China	2016
OP699292	I	Swine	Guangxi, China	2016
OP699293	I	Swine	Guangxi, China	2016
OP699294	I	Swine	Guangxi, China	2016
OP699295	I	Swine	Heilongjiang, China	2018
OP699296	I	Swine	Heilongjiang, China	2018
OP699297	I	Swine	Heilongjiang, China	2018
OP699298	I	Swine	Heilongjiang, China	2018
OP699299	I	Swine	Heilongjiang, China	2018
OP699300	I	Swine	Jilin, China	2018
OP699301	I	Swine	Jilin, China	2018

**Table 2:** The amino acids of proteins E in JEV strains.

	76th Site	95th Site	123rd Site	138th Site	244th Site	474th Site	475th Site
consensus	T	G	S	E	E	N	A
OP699289			S-N				A-K
OP699290	T-M				E-Q		A-K
OP699291	T-M				E-Q		A-K
OP699292			S-N	E-K			A-K
OP699293			S-N	E-K			A-K
OP699294			S-N	E-K			A-K
OP699295		G-D	S-N	E-K			A-K
OP699296			S-N	E-K			A-K
OP699297			S-N	E-K			A-K
OP699298			S-N	E-K		N-K	A-R
OP699299			S-N	E-K			A-K
OP699300			S-N	E-K			A-K
OP699301	T-M				E-Q		A-K

**Table 3:** Identical phosphorylation sites in JEV E protein.

Site	Kinase	Score	Site	Kinase	Score	Site	Kinase	Score
40 T	PKC	0.591	210 S	unsp	0.787	344 S	unsp	0.966
51 S	DNAPK	0.564	222 S	cdc2	0.532	349 T	p38MAPK	0.558
66 T	PKC	0.662	222 S	DNAPK	0.531	349 T	cdk5	0.524
70 T	PKC	0.824	222 S	PKA	0.518	356 T	PKC	0.566
88 S	unsp	0.985	226 T	GSK3	0.504	363 T	PKC	0.693
89 S	PKC	0.816	226 T	PKC	0.501	364 S	unsp	0.944
90 Y	unsp	0.950	227 S	unsp	0.945	365 S	PKC	0.762
112 S	PKA	0.545	227 S	cdk5	0.548	368 S	unsp	0.734
115 T	PKC	0.749	230 S	PKC	0.586	382 Y	unsp	0.973
147 T	unsp	0.665	257 S	DNAPK	0.621	382 Y	INSR	0.553
148 T	unsp	0.627	257 S	unsp	0.532	401 S	PKC	0.773
148 T	PKC	0.548	277 S	ATM	0.502	401 S	cdc2	0.565
162 S	PKC	0.666	277 S	PKC	0.818	402 T	PKC	0.786
162 S	DNAPK	0.604	277 S	unsp	0.528	408 S	PKC	0.62
162 S	ATM	0.539	281 T	PKC	0.575	408 S	cdc2	0.518
168 T	PKG	0.577	282 S	PKC	0.507	409 T	cdc2	0.537
183 Y	EGFR	0.617	301 Y	SRC	0.518	410 T	unsp	0.854
183 Y	unsp	0.599	305 T	PKC	0.669	410 T	PKC	0.737
187 T	unsp	0.738	309 S	PKC	0.717	429 S	CKI	0.618
194 S	unsp	0.954	317 T	unsp	0.933	460 T	DNAPK	0.554
202 Y	unsp	0.655	327 T	CKI	0.569	460 T	cdc2	0.55
205 T	PKC	0.853	329 S	unsp	0.994			

Score: the prediction score (a value in the range [0.000–1.000]; the scores above 0.500 indicate positive predictions). Kinase: the active kinase or the string “unsp” for non-specific prediction.

## Data Availability

Not applicable.

## References

[1] Han N., Adams J., Chen P., Guo Z.-Y., Zhong X.-F., Fang W., Li N., Wen L., Tao X.-Y., Yuan Z.-M. (2014). Comparison of Genotypes I and III in Japanese Encephalitis Virus Reveals Distinct Differences in Their Genetic and Host Diversity. J. Virol..

[2] Gao X., Liu H., Li M., Fu S., Liang G. (2015). Insights into the evolutionary history of Japanese encephalitis virus (JEV) based on whole-genome sequences comprising the five genotypes. Virol. J..

[3] Schuh A.J., Guzman H., Tesh R.B., Barrett A.D. (2013). Genetic diversity of Japanese encephalitis virus isolates obtained from the Indonesian archipelago between 1974 and 1987. Vector-Borne Zoonotic Dis..

[4] Do L.P., Bui T.M., Phan N.T. (2016). Mechanism of Japanese encephalitis virus genotypes replacement based on human, porcine and mosquito-originated cell lines model. Asian Pac. J. Trop. Med..

[5] Huang Y.S., Hettenbach S.M., Park S.L., Higgs S., Barrett A.D., Hsu W.W., Harbin J.N., Cohnstaedt L.W., Vanlandingham D.L. (2016). Differential Infectivities among Different Japanese Encephalitis Virus Genotypes in *Culex quinquefasciatus* Mosquitoes. PLoS Negl. Trop. Dis..

[6] Auerswald H., Maquart P.O., Chevalier V., Boyer S. (2021). Mosquito Vector Competence for Japanese Encephalitis Virus. Viruses.

[7] Simpson D.I., Way H.J., Platt G.S., Bowen E.T., Hill M.N., Kamath S. (1975). Arbovirus infections in Sarawak, October 1968–February 1970: GETAH virus isolations from mosquitoes. Trans. R. Soc. Trop. Med. Hyg..

[8] Ladreyt H., Durand B., Dussart P., Chevalier V. (2019). How Central is the Domestic Pig in the Epidemiological Cycle of Japanese Encephalitis Virus? A Review of Scientific Evidence and Implications for Disease Control. Viruses.

[9] Le Flohic G., Porphyre V., Barbazan P., Gonzalez J.P. (2013). Review of climate, landscape, and viral genetics as drivers of the Japanese encephalitis virus ecology. PLoS Negl. Trop. Dis..

[10] Ricklin M.E., Garcia-Nicolas O., Brechbuhl D., Python S., Zumkehr B., Posthaus H., Oevermann A., Summerfield A. (2016). Japanese encephalitis virus tropism in experimentally infected pigs. Vet. Res..

[11] Mansfield K.L., Hernandez-Triana L.M., Banyard A.C., Fooks A.R., Johnson N. (2017). Japanese encephalitis virus infection, diagnosis and control in domestic animals. Vet. Microbiol..

[12] Ladreyt H., Auerswald H., Tum S., Ken S., Heng L., In S., Lay S., Top C., Ly S., Duong V. (2020). Comparison of Japanese Encephalitis Force of Infection in Pigs, Poultry and Dogs in Cambodian Villages. Pathogens.

[13] Peiris J.S., Amerasinghe F.P., Arunagiri C.K., Perera L.P. (1993). Japanese encephalitis in Sri Lanka: Comparison of vector and virus ecology in different agro-climatic areas. Trans. R. Soc. Trop. Med. Hyg..

[14] Zhu Y., Xu J., Lian S., Zhang R., Hou J., Wang M., Yan X. (2022). Difference Analysis Between Canine Adenovirus Types 1 And 2. Front. Cell. Infect. Microbiol..

[15] Sun Y., Ding H., Zhao F., Yan Q., Li Y., Niu X., Zeng W., Wu K., Ling B., Fan S. (2022). Genomic Characteristics and E Protein Bioinformatics Analysis of JEV Isolates from South China from 2011 to 2018. Vaccines.

[16] Zhu Y., Sun J., Yan M., Lian S., Hu B., Lv S., Li Y., Zhang Y., Yan X. (2021). The biological characteristics of the canine adenovirus type 1 from fox and the transcriptome analysis of the infected MDCK cell. Cell Biol. Int..

[17] Schuh A.J., Ward M.J., Leigh Brown A.J., Barrett A.D. (2014). Dynamics of the emergence and establishment of a newly dominant genotype of Japanese encephalitis virus throughout Asia. J. Virol..

[18] Gao X., Liu H., Wang H., Fu S., Guo Z., Liang G. (2013). Southernmost Asia is the source of Japanese encephalitis virus (genotype 1) diversity from which the viruses disperse and evolve throughout Asia. PLoS Negl. Trop. Dis..

[19] Schuh A.J., Ward M.J., Brown A.J., Barrett A.D. (2013). Phylogeography of Japanese encephalitis virus: Genotype is associated with climate. PLoS Negl. Trop. Dis..

[20] Boyer S., Durand B., Yean S., Brengues C., Maquart P.O., Fontenille D., Chevalier V. (2021). Host-Feeding Preference and Diel Activity of Mosquito Vectors of the Japanese Encephalitis Virus in Rural Cambodia. Pathogens.

[21] Bhattachan A., Amatya S., Sedai T.R., Upreti S.R., Partridge J. (2009). Japanese encephalitis in hill and mountain districts, Nepal. Emerg. Infect. Dis..

[22] Zhang H., Luo H., Rehman M.U., Nabi F., Li K., Lan Y., Huang S., Zhang L., Mehmood K., Shahzad M. (2017). Evidence of JEV in Culex tritaeniorhynchus and pigs from high altitude regions of Tibet, China. J. Vector Borne Dis..

[23] Impoinvil D.E., Baylis M., Solomon T. (2013). Japanese encephalitis: On the One Health agenda. Curr. Top. Microbiol. Immunol..

[24] Lindahl J., Chirico J., Boqvist S., Thu H.T.V., Magnusson U. (2012). Occurrence of Japanese encephalitis virus mosquito vectors in relation to urban pig holdings. Am. J. Trop. Med. Hyg..

[25] Lardeux F., Loayza P., Bouchite B., Chavez T. (2007). Host choice and human blood index of *Anopheles pseudopunctipennis* in a village of the Andean valleys of Bolivia. Malar. J..

[26] Lin C.W., Wu S.C. (2003). A functional epitope determinant on domain III of the Japanese encephalitis virus envelope protein interacted with neutralizing-antibody combining sites. J. Virol..

[27] Gromowski G.D., Firestone C.Y., Whitehead S.S. (2015). Genetic Determinants of Japanese Encephalitis Virus Vaccine Strain SA14-14-2 That Govern Attenuation of Virulence in Mice. J. Virol..

[28] Yang J., Yang H., Li Z., Wang W., Lin H., Liu L., Ni Q., Liu X., Zeng X., Wu Y. (2017). Envelope Protein Mutations L107F and E138K Are Important for Neurovirulence Attenuation for Japanese Encephalitis Virus SA14-14-2 Strain. Viruses.

[29] Yang D., Li X.F., Ye Q., Wang H.J., Deng Y.Q., Zhu S.Y., Zhang Y., Li S.H., Qin C.F. (2014). Characterization of live-attenuated Japanese encephalitis vaccine virus SA14-14-2. Vaccine.

[30] Su H.L., Liao C.L., Lin Y.L. (2002). Japanese encephalitis virus infection initiates endoplasmic reticulum stress and an unfolded protein response. J. Virol..

[31] Zhang Y., Chen P., Cao R., Gu J. (2011). Mutation of putative N-linked glycosylation sites in Japanese encephalitis virus premembrane and envelope proteins enhances humoral immunity in BALB/C mice after DNA vaccination. Virol. J..

[32] Dechtawewat T., Roytrakul S., Yingchutrakul Y., Charoenlappanit S., Siridechadilok B., Limjindaporn T., Mangkang A., Prommool T., Puttikhunt C., Songprakhon P. (2021). Potential Phosphorylation of Viral Nonstructural Protein 1 in Dengue Virus Infection. Viruses.

